# Flow-Induced Dynamic Dispersion in Dispersant-Free Mixed-Oxide Slurry Systems

**DOI:** 10.1021/acs.langmuir.5c05380

**Published:** 2026-02-13

**Authors:** Yu-An Lin, Feng-Ming Yeh, Bin Hu, Ting-Kai Huang, Hsin-Hsien Lu, Hong Zhong, Chia-Chen Li

**Affiliations:** † Department of Materials Science and Engineering, 34881 National Tsing Hua University, Hsinchu 30013, Taiwan; ‡ CMP Division, 477069 FUJIFILM Electronic Materials U.S.A., Inc., Mesa, Arizona 85212, United States; § CMP Division, FUJIFILM Electronic Materials Taiwan Co., Ltd., Hsinchu County 303036, Taiwan

## Abstract

This study demonstrates that a particle-mixing strategy in aqueous suspension is more effective than chemical dispersants in enhancing the dynamic dispersion and performance of SiO_2_-based slurries for planarization applications. By preparing particle-mixed suspensions containing 25 and 55 nm SiO_2_ particles at chemical-mechanical planarization (CMP)-relevant solid loadings (1–10 wt %), we show that combining these two particle sizes suppresses agglomeration and transforms the suspension rheology from shear-thinning to a nearly Newtonian response under flow, indicating improved dynamic dispersion after yielding. Small-angle X-ray scattering and effective volume packing analyses confirm that cooperative size effects drive the improved structural organization, thereby enhancing flow behavior. In contrast, the commonly used ammonium polyacrylate dispersant enhances static dispersion but fails to produce uniform flow behavior under shear. In CMP tests, suspensions with a bimodal particle size distribution achieve higher material removal rates and lower surface roughness than monodisperse or dispersant-stabilized suspensions simultaneously. Numerical simulations that couple the discrete element method and computational fluid dynamics further show that the improved CMP performance, resulting from the use of the powder-mixing suspension, is due to denser particle contacts and higher localized stresses in the bimodal system.

## Graphical abstract

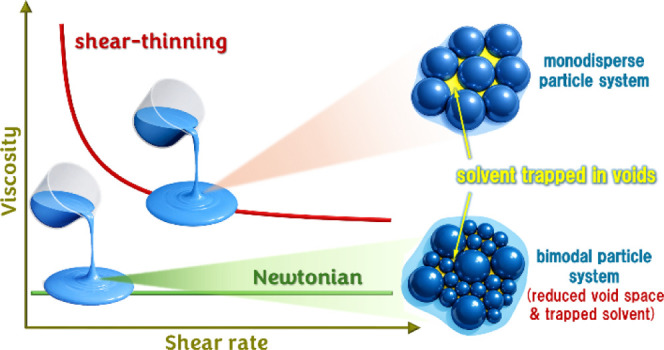

## Introduction

The rheological behavior of silica-based suspensions has been extensively investigated due to their relevance in both fundamental colloid science and industrial processes such as chemical mechanical planarization (CMP). In particular, suspensions composed of spherical and fumed silica particles exhibit markedly different flow behaviors owing to their distinct morphologies and aggregation tendencies. Spherical silica particles are often treated as model hard-sphere systems, whereas fumed silicas consist of branched, fractal-like aggregates that readily form percolated networks under quiescent and shear conditions. As a result, fumed silica suspensions frequently display pronounced shear thinning, shear thickening, or even irreversible rheological transitions at relatively low to moderate solid loadings.^1^ Recent rheological studies have demonstrated that such non-Newtonian responses are governed not only by particle size but also by particle concentration, packing density, and the formation and disruption of transient contact networks under shear. For example, introducing spherical silica particles into fumed silica suspensions can effectively suppress irreversible shear thickening by disrupting aggregate connectivity, underscoring the importance of geometric and microstructural control in addition to surface chemistry.^1^

Beyond particle morphology, particle size asymmetry and size ratio effects play a crucial role in determining interparticle interactions and suspension stability.^2^ Studies on microsphere-nanoparticle mixtures have revealed that the addition of smaller particles can fundamentally alter interparticle forces through mechanisms such as nanoparticle halo formation, depletion effects, or nanoparticle-mediated bridging, depending on size ratio, surface charge, and ionic strength.^3–6^ It has been demonstrated that nanoparticle haloing can stabilize otherwise flocculating microspheres over a finite window of nanoparticle volume fractions,^4^ while both theoretical and experimental studies have further shown that even dilute nanoparticle additives can induce substantial changes in suspension elasticity and flow response by modifying local microstructure and stress transmission pathways. These findings collectively highlight that bimodal or multimodal particle systems offer a powerful route to tune suspension rheology through physical structuring effects, rather than relying solely on chemical dispersants.

In the context of CMP, these considerations are particularly critical.^7^ CMP is a key planarization process in advanced semiconductor manufacturing,^8–10^ enabling the fabrication of multilayer interconnects and high-density device architectures through the synergistic action of chemical reactions and mechanical abrasion. The CMP slurry, which consists of abrasive particles dispersed in a chemically active liquid, plays a central role in determining both material removal efficiency and surface quality.^11–13^ However, slurry optimization has long been constrained by a fundamental trade-off: increasing the material removal rate (MRR) often leads to higher surface roughness and defect density, whereas strategies aimed at minimizing surface damage typically reduce polishing throughput.^14–16^ Conventional CMP slurry formulations frequently rely on chemical dispersants to suppress particle agglomeration and stabilize suspensions.^17–20^ While effective to some extent, these additives can alter particle surface chemistry, introduce organic residues, and complicate postpolishing cleaning and interfacial control, issues that are particularly undesirable for high-purity semiconductor processes.^21–23^ To address these challenges, alternative approaches such as tuning particle size distributions, modifying abrasive compositions, or introducing hybrid abrasive systems have been explored.^24–28^ Nevertheless, the mechanistic links between slurry microstructure, rheological behavior under dynamic flow, and polishing performance remain incompletely understood.

A key limitation of many prior studies is their reliance on quiescent characterization techniques, such as particle size distribution measurements or sedimentation tests, which provide limited insight into the dynamic particle arrangements that arise during polishing. Under CMP conditions, slurries experience complex shear fields, and the dispersion state can evolve rapidly in response to flow. In particular, slurries containing monodisperse abrasives often exhibit pronounced shear-thinning behavior, reflecting the formation and disruption of weak, shear-sensitive agglomerates. Such transient microstructures can lead to unstable removal rates and increased risk of surface scratching. These limitations underscore the need for slurry designs that achieve not only chemical cleanliness but also intrinsic dynamic stability under shear.

Accordingly, this study advances a dispersant-free strategy for CMP slurry design by emphasizing a simple yet highly effective concept: targeted particle-size mixing. While the use of bimodal or multimodal particle size distributions to improve dispersion and packing efficiency has been recognized in colloid science, its systematic implementation as a viable alternative to chemical dispersants in CMP slurries remains relatively unexplored. Here, we hypothesize that constructing a bimodal particle size distribution through the controlled mixing of two monodisperse oxide particle populations can fundamentally improve slurry microstructure and flow behavior without relying on chemical additives.^29–34^ To test this hypothesis, we establish a clear mechanistic framework linking multiscale particle packing to dynamic rheological response and, ultimately, to polishing performance. This is achieved through a comprehensive experimental-computational approach that integrates synchrotron small-angle X-ray scattering (SAXS) to probe slurry microstructure, rheological measurements to characterize flow behavior under shear, and CMP polishing tests interpreted with the aid of coupled computational fluid dynamics and discrete element method (CFD-DEM) simulations.^35–38^ Through this combined analysis, we demonstrate that particle-size mixing effectively suppresses shear-sensitive aggregation, enhances flow uniformity, and enables simultaneous improvement in MRR and surface quality. These findings provide practical insight into packing- and flow-governed mechanisms and offer clear design guidelines for high-purity, chemically clean slurry formulations for advanced CMP applications.

## Materials and Methods

### Raw Materials

Two SiO_2_ nanopowders with median sizes of 55 nm (99.5%, Sigma-Aldrich, USA) and 25 nm (>99.5%, SEED CHEM, Australia) were used as abrasives to prepare aqueous suspensions. Ammonium poly­(acrylic acid) (PAA-NH_4_; Darvan-821A, Vanderbilt Minerals, LLC in Norwalk, USA), with a molecular weight of 3000 g mol^–1^, was used as the dispersant, and deionized water served as the dispersion medium.

### Characterizations

The SiO_2_ nanopowders were characterized using a transmission electron microscope (TEM; HT7700, Hitachi, Japan) and X-ray photoelectron spectroscopy (XPS; PHI 5000, Versaprobe II, ULVAC-PHI Inc., Japan). For the following experiments, the aqueous suspensions were prepared by dispersing nano-SiO_2_ particles with or without PAA-NH_4_ (160 mg g^–1^ SiO_2_), with the pH adjusted and maintained at 10 using sodium hydroxide solution. To deagglomerate the nanopowders, the slurries were ball-milled (MUBM236, TOHAMA Co., Ltd., Taiwan) at 180 rpm for a fixed duration of 24 h using 5 mm-diameter yttria-stabilized zirconia milling balls, with a ball-to-slurry volume ratio of approximately 1:1. This provides sufficient impact and shear forces for deagglomeration while preventing excessive ball–ball collisions that could cause milling media wear and slurry contamination. Aqueous slurries containing 10 wt % SiO_2_ particles were used for sedimentation height measurements and particle size analysis via dynamic light scattering (DLS; SZ-100, Horiba, Japan). Before particle size measurements, the slurries were diluted to 0.1 wt %. For zeta potential analysis and planarization tests, slurries with 1 wt % SiO_2_ were prepared, and the zeta potential was measured using an electroacoustic method (ZetaProbe, Colloidal Dynamics Inc., North Attleborough, MA, USA). Rheological properties were evaluated using a commercial rotational rheometer (MCR302e, Anton Paar, Austria) with slurries containing 5 wt % SiO_2_ using a double-gap coaxial cylinder geometry. Flow curves were acquired under steady-state conditions using stepwise shear-rate control, with each shear rate maintained until a constant viscosity response was achieved before recording. For adsorption measurements of PAA-NH_4_ on SiO_2_, aqueous suspensions containing 5 wt % SiO_2_ were mixed with varying concentrations of PAA-NH_4_ by ball milling at 180 rpm for 24 h. After equilibration, the suspensions were centrifuged at 11,000 rpm for 15 min and subsequently filtered to collect the supernatants. The residual PAA-NH_4_ concentration in the supernatant was quantified by potentiometric titration, and the amount of adsorbed PAA-NH_4_ was determined using a mass-balance calculation. Details of this analytical procedure have been reported previously.^39^

Synchrotron SAXS measurements were conducted at beamline TLS 23A1 (National Synchrotron Radiation Research Center, Taiwan) using a monochromatic X-ray beam (λ = 1.55 Ȧ) with a sample-to-detector distance of 1 m, covering a *q*-range of 0.002–1 Ȧ^–1^. Slurries were measured under both static and oscillatory shear conditions. For shear measurements, an oscillatory shear cell was employed and operated at a low stress amplitude (0.1 Pa) and a low-frequency oscillatory shear (1 Hz), corresponding to the linear viscoelastic regime. Rheo-SAXS measurements were performed in situ during oscillatory deformation. Prior to data acquisition, samples were allowed to equilibrate at rest to ensure a reproducible initial microstructural state. SAXS patterns were collected after the scattering signal reached a steady and reproducible state under oscillatory shear. The SAXS acquisition time for each measurement was short compared to the oscillation period, such that the resulting patterns represent time-averaged microstructural configurations under oscillatory shear. The resulting two-dimensional (2D) SAXS patterns were azimuthally averaged to obtain one-dimensional (1D) intensity profiles and were subsequently background-subtracted for analysis.

For the planarization tests, Si wafer pieces (4 × 4 cm) with an initial surface roughness (Rq) of 4.0 ± 0.1 nm were used as the polishing samples. Surface roughness was characterized using an atomic force microscope (AFM; Dimension ICON, Bruker, USA) operated in tapping mode. AFM measurements were performed using silicon cantilevers with a nominal spring constant of ∼40 N m^–1^ and a resonance frequency of ∼300 kHz. Prior to imaging, the cantilever was tuned by frequency sweep to determine the resonance frequency and quality factor, and the spring constant was calibrated using the thermal noise method. All AFM measurements were conducted under ambient conditions, and surface roughness values were extracted from height images after standard plane correction. Polishing was performed on a CMP machine (HK-DP-182, Hokang S.M. Co., Taiwan) at a rotation speed of 100 rpm for a specified duration. The material removal rate (MRR) was calculated as MRR = (mass loss)/(Si density × polished area × polishing time).^40^

### Numerical Simulations

A numerical simulation using a two-way coupling model, combining DEM and CFD, was performed. The DEM was executed with Ansys Rocky 2023R2 software, and CFD was done using Ansys Fluent 2023R2.^35^ The DEM calculated the movement of particles based on Newton’s second law of motion, while CFD analyzed the behavior of the fluid phase by creating a numerical grid to represent the fluid body and its boundaries. The relative parameters used in the calculations were also provided (Table S1, Supporting Information).

## Results and Discussion

### Mixing Effect on Dispersion

The two SiO_2_ nanopowders used to prepare the aqueous suspensions have primary sizes of 25 and 55 nm, as shown in their TEM images in Figure 1a,b. Although both powders tend to form agglomerates, which is more noticeable for the smaller 25 nm SiO_2_ in Figure 1b, their particles are highly uniform in size. In this study, the solid loading of the SiO_2_ suspensions was fixed at 10 wt %, a concentration selected to be sufficiently high to sensitively probe agglomeration and reagglomeration behavior, while remaining relevant to practical slurry formulations and ensuring stable processing. Importantly, this solid content is representative of commercially available CMP slurries, which are typically supplied at moderate solid loadings and subsequently diluted by end users to meet specific process requirements. After 24 h of ball milling, agglomeration persists, with median sizes of 38 and 69 nm for the 25 and 55 nm SiO_2_, respectively (Figure 1c,d, solid lines). When the two SiO_2_ nanopowders are mixed in a 1:1 volume ratio and ball-milled, a bimodal particle distribution appears, with peaks near the primary sizes of 25 and 55 nm for each one, as seen in Figure 1e (solid line). This indicates that powder mixing enhances deagglomeration efficiency. Since this effect is crucial for producing a well-dispersed slurry without dispersants, we further studied how the two SiO_2_ powders interact in suspension by examining their reagglomeration behavior over time, surface charge properties, surface chemistry, and other relevant features that could reveal the underlying mechanism.

**Figure 1:**
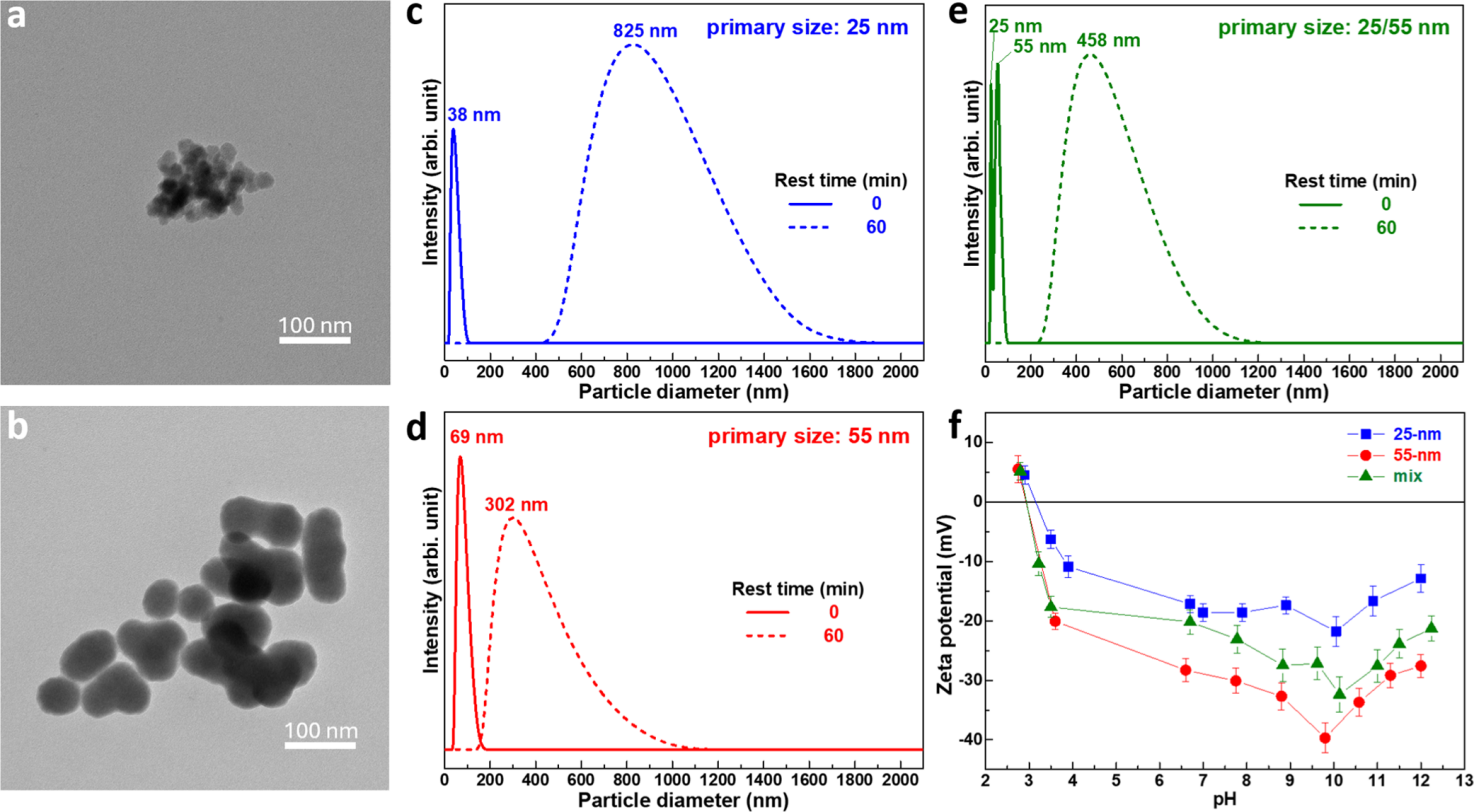
TEM images of the as-received SiO_2_ nanoparticles with primary sizes of (a) 25 nm and (b) 55 nm. Particle size distributions of 10 wt % aqueous suspensions containing (c) solely 25 nm, (d) solely 55 nm, and (e) mixed SiO_2_ particles after ball milling at 180 rpm for 24 h (solid lines) and after reagglomeration following 1 h of resting (dashed lines). (f) Zeta potentials of 1 wt % suspensions of 25 nm, 55 nm, and mixed SiO_2_ particles. All particle size distribution measurements (c–e) were conducted at an equilibrium pH of 10.

The reagglomeration behavior of the nanopowders in aqueous suspension was assessed by monitoring particle size during a 1 h resting period, as shown in Figure 1c–e (Figure S1, Supporting Information). After 1 h, the median size of the 25 nm SiO_2_ increased from 38 to 825 nm (Figure 1c, dashed line), whereas the 55 nm SiO_2_ grew from 69 to 302 nm (Figure 1d, dashed line), consistent with a higher tendency for smaller particles to form agglomerates. In the mixed suspension, the initially bimodal distribution evolved into a single dominant peak centered at an agglomerated size of ∼458 nm (Figure 1e, dashed line). It should be noted that, due to the intensity-weighted nature of DLS measurements, this peak primarily reflects the dominant population of large agglomerates formed during rest and, within the resolution of DLS, is consistent with collective aggregation of particles rather than size-selective segregation. To clarify why the 55 nm SiO_2_ undergoes less reagglomeration, zeta potential measurements were performed. It should be emphasized that particle size distributions discussed above were determined by DLS, whereas the zeta potential data presented below were measured independently using an electroacoustic method; these two techniques probe different physical interactions and serve complementary roles in this study. As shown in Figure 1f, the 55 nm particles have a substantially higher zeta potential magnitude than the 25 nm particles, providing stronger electrostatic repulsion that mitigates the tendency for agglomeration. Since both powders are nanosized SiO_2_, XPS analysis was conducted to investigate potential surface chemistry differences that may account for this disparity. The results (Figure S2, Supporting Information) showed similar overall compositions, but the 55 nm SiO_2_ contained a slightly higher fraction of surface Si–OH groups, which may contribute to its greater pH sensitivity and higher zeta potential.

In the mixed system containing 25 and 55 nm SiO_2_ particles at a 1:1 volume ratio, the measured zeta potential falls between those of the two monodisperse suspensions. It is recognized that electroacoustic zeta potential measurements represent an effective, population-averaged response weighted by particle mobility, which depends on particle size, density, and concentration.^41^ Despite the higher number density of the 25 nm particles, their contribution does not dominate the electroacoustic response of the mixture. A plausible explanation is that the finer particles in the monodisperse 25 nm suspension are more prone to reagglomeration, which can reduce the apparent zeta potential measured by electroacoustic methods due to aggregation-induced attenuation of effective interfacial mobility. Upon mixing with the 55 nm particles, the extent of fine–fine reagglomeration during rest is mitigated, leading to an improved dispersion state of the smaller particles. This interpretation is supported by Figure 1e (dashed line), where the mixed suspension exhibits a smaller dominant reagglomerated size (∼458 nm) than the monodisperse 25 nm suspension (∼825 nm) after 60 min. Such moderated reagglomeration is expected to enhance the effective exposure of particle surfaces to the aqueous medium, thereby facilitating greater dissociation of surface Si–OH groups,^41,42^ and resulting in a more negative apparent zeta potential in the mixed system compared with the monodisperse 25 nm suspension. At the same time, because electroacoustic measurements yield a mobility-weighted effective zeta potential^43^ and the 55 nm particles inherently exhibit more negative surface potentials, their contribution further shifts the overall response toward more negative values. Consequently, the zeta potential of the mixed system remains intermediate between those of the individual suspensions. Consistently, the order of zeta potential magnitudes, 55 nm SiO_2_ > mixed particles >25 nm SiO_2_, correlates well with the reagglomerated sizes shown in Figure 1e (dashed line).

### Quiescent Dispersion Stability

In addition to particle size analysis, the quiescent dispersion stability of the three suspensions was assessed through sedimentation experiments. As shown in Figure 2a, during the first 2 min of settling, the sedimentation height of all three systems increases linearly with time, indicating a nearly constant settling velocity. From the slopes, the 25 nm SiO_2_ exhibits the highest velocity (5.3 × 10^–7^ m s^–1^), the 55 nm SiO_2_ the lowest (7.5 × 10^–8^ m s^–1^), and the mixed system an intermediate value (2.5 × 10^–7^ m s^–1^). This linear behavior satisfies the assumption of Stokes’ law, allowing the average particle radius (*r*) to be estimated from the terminal velocity (*v*_t_) using eq 1 ^44^

1 $$v_{t}=\frac{2}{9}\;\frac{\left(\rho_{s}-\rho_{f}\right)\;gr^{2}}{\eta }$$

where ρ_s_ and ρ_f_ are the solid and fluid densities, respectively, η is the fluid viscosity, and *g* is gravitational acceleration. The calculated particle diameters are 878, 328, and 608 nm for the 25 nm, 55 nm, and mixed systems, respectively, which are consistent with the reagglomerated sizes shown in Figure 1c–e. The higher sedimentation height of the 25 nm SiO_2_ should correspond to its larger agglomerates, which result in looser particle packing than in the mixed and 55 nm systems. Figure 2b shows photographs of the suspensions after 108 h of rest; all remain cloudy with indistinct phase boundaries, suggesting slow sedimentation of the nanopowders.

**Figure 2:**
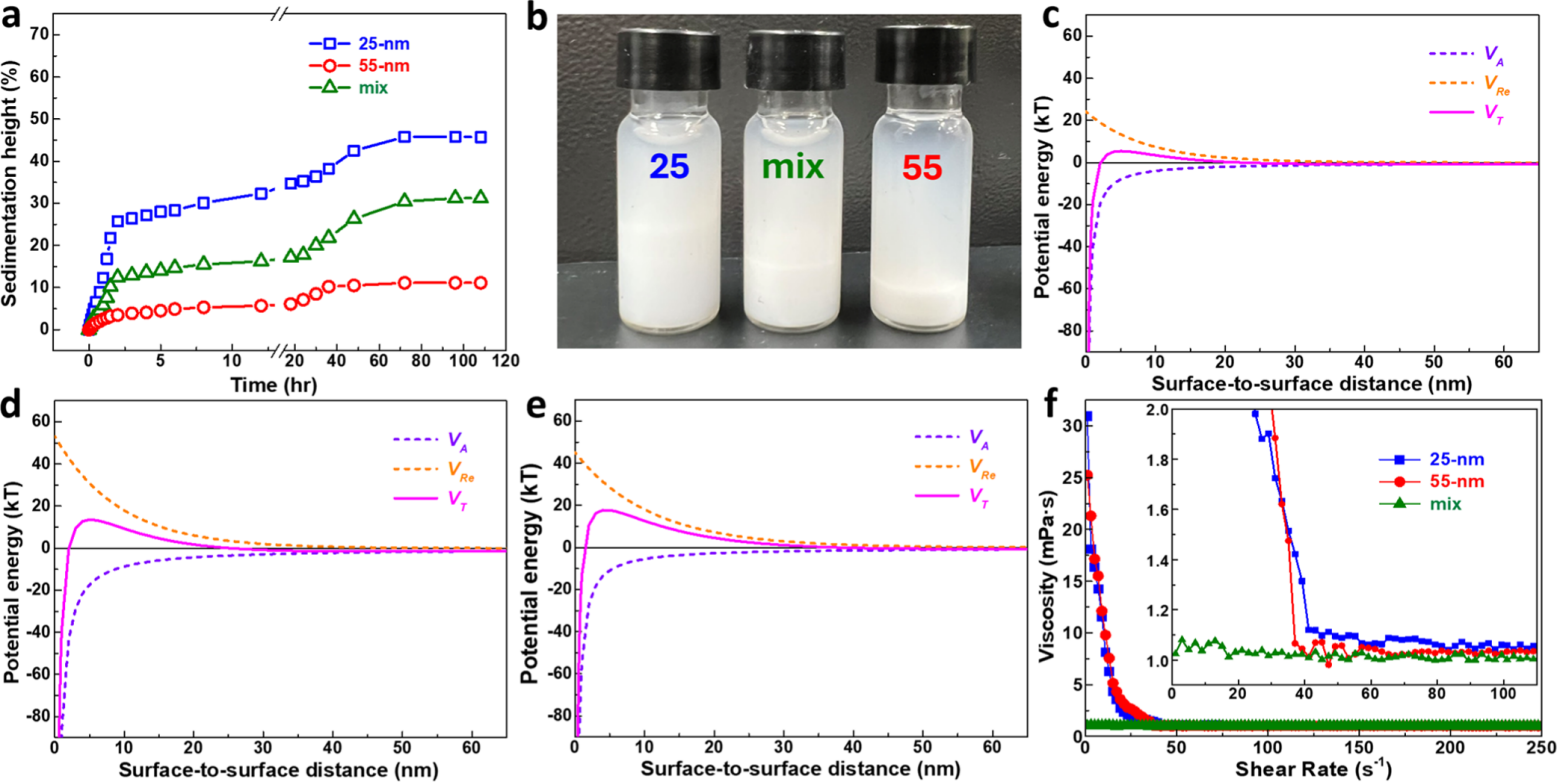
(a) Sedimentation height and (b) corresponding photographs of aqueous suspensions (10 wt % powder content) after resting for 108 h with 25 nm, 55 nm, and mixed SiO_2_ particles. Potential energies of (c) 25 nm, (d) 55 nm, and (e) mixed SiO_2_ particles in aqueous suspensions. (f) Viscosity as a function of shear rate for aqueous suspensions containing 5 wt % 25 nm, 55 nm, and mixed SiO_2_ particles (inset: magnified view). All aqueous suspensions were measured at an equilibrium pH of 10.

The improved dispersion of the two SiO_2_ nanopowders in the mixed-particle suspension can be further supported by DLVO-based calculations,^40,44–47^ showing a higher energy barrier for agglomeration. In the calculations, the van der Waals attraction (*V*_*A*_) between spherical SiO_2_ particles is assumed to be the sole attractive force and is given by

2 $$V_{A}=-\frac{A_{121}}{6H}\left(\frac{r_{1}r_{2}}{r_{1}+r_{2}}\right)$$

where *H* is the interparticle separation (*H* < *r*_1_ and *H* < *r*_2_), *r*_1_ and *r*_2_ are particle radii, and *A*_121_ is the Hamaker constant of the dispersion system. *A*_121_ can be determined from the Hamaker constants of the particles (*A*_11_) and solvent (*A*_22_) (Table 1) using^41,45,46^

3 $$A_{121}={\left(\sqrt{A_{11}}-\sqrt{A_{22}}\right)}^{2}$$

In addition to attraction, the main repulsive force in aqueous SiO_2_ suspensions is the electrostatic repulsion (*V*_re_), expressed as

4 $$V_{re}=\frac{2\pi r\varepsilon_{r}\varepsilon_{0}\psi_{0}^{2}\ln\left(1+e^{-\kappa h}\right)}{kT}$$

where ε_r_ and ε_0_ are the dielectric constants of the solvent and vacuum, respectively. ψ_0_ is the particle surface potential (approximated by the zeta potential, ζ), *k* is the Boltzmann’s constant, and *T* is the temperature. The reciprocal of the electrical double-layer thickness, κ, is given by

5 $$\kappa ={\left(\frac{\varepsilon_{r}kT}{8\pi ne^{2}Z^{2}}\right)}^{-0.5}$$

where *n* is the ion concentration in the suspension and *Z* is the ion valence. The total interaction energy profiles (*V*_*T*_) of the three suspensions are shown in Figure 2c–e. The 25 nm system (Figure 2c) exhibits the weakest repulsion, yielding the lowest barrier (∼8 kT). In the 55 nm suspension (Figure 2d), the attractive interaction is stronger, but the higher zeta potential enhances repulsion, producing a larger barrier (∼17–18 kT). In the mixed suspension (Figure 2e), the attractive energy is comparable to that of the 25 nm system, while the repulsive energy approaches that of the 55 nm system. As a result, the barrier (∼19–20 kT) is slightly higher than in the 55 nm case, consistent with the reduced agglomeration observed in Figure 1e (dashed line).

**Table 1:** Parameters Required for DLVO Calculations

	SiO_2_ nanopowder		
parameters	25 nm	mix	55 nm
*a* (nm)	12.5		27.5
*A*_11_ (J)		7.8 × 10^–20^	
*A*_22_ (J)		3.8 × 10^–20^	
*A*_121_ (J)		7.11 × 10^–21^	

### Dynamic Dispersion Property

Beyond quiescent dispersion properties, the rheological behavior of suspensions under flow, which demonstrates the “dynamic” dispersion state, is even more critical for most slurry applications, such as CMP processes. Figure 2f compares the viscosity-shear rate profiles of aqueous suspensions containing only 25 nm, 55 nm, or mixed SiO_2_ particles. The two monodisperse systems exhibit pronounced shear-thinning behavior: viscosity decreases sharply with increasing shear rate before leveling off at a low steady value at higher rates.^44^ According to colloidal dispersion theory, the flow behavior of suspensions can be characterized by a rate index (*n*), which is obtained by fitting the rheological curves of suspensions with the Herschel-Bulkley model, as shown in eq 6 ^44,47^

6 $$\tau =\tau_{y}+{\mathrm{\gamma ̇}}^{n}$$

where τ is the shear stress, τ_*y*_ is the yield stress, and γ̇ is the shear rate. For the suspensions, obtaining an *n* value of 1.0 indicates Newtonian flow, those with an *n* value of <1.0 a shear-thinning flow, and those with an *n* value of >1 a shear-thickening flow. Here, the flow behavior index *n* is used as a phenomenological indicator to distinguish non-Newtonian (e.g., shear-thinning or shear-thickening) from Newtonian flow, rather than as a statistically averaged quantity. The resulting *n* values for the aqueous suspensions of only 25 nm, 55 nm, and mixed SiO_2_ particles are 0.65, 0.72, and 1.01, respectively. Clearly, the two monodisperse systems exhibit shear-thinning behavior, indicating the presence of soft particle agglomerates in the suspensions. The decreasing viscosity with increasing shear rate is due to the progressive disruption of these agglomerates by shear stress, resulting in a more deagglomerated and freely flowing suspension (Figure S3 and Video S1, Supporting Information). This rheological characteristic is consistent with the microstructural observations in Figure 1c,d (solid lines), where residual agglomerates remain after ball milling.

In contrast, the mixed-particle suspension exhibits a predominantly Newtonian flow behavior, with viscosity remaining nearly constant across the entire range of applied shear rates. Such reproducible rheological characteristics strongly suggest that the particles in the mixed system remain uniformly dispersed, even under low shear conditions where flocculation would typically occur in single-size suspensions. This stability reflects the inherently weak effective interparticle interactions in the mixed configuration, arising from the optimized bimodal packing of particles of different sizes.^32,48,49^ In this arrangement, larger particles form a loose structural framework while smaller particles occupy interstitial spaces, reducing the probability of sustained particle–particle contacts and suppressing the formation of stress-bearing agglomerated networks.^50^ As a result, hydrodynamic lubrication dominates over direct contact interactions, leading to a reduced sensitivity of the suspension microstructure to applied shear.^51^ This interpretation is further consistent with the reduced particle size distribution obtained after ball milling, as shown in Figure 1e (solid line), confirming that the mixed-particle strategy effectively minimizes persistent interactions within soft agglomerates and allows them to readily disintegrate under flow, thereby producing a stable Newtonian-like dynamic dispersion.^52^

### Mechanism Behind Improved Dynamic Dispersion

Regarding the Newtonian flow behavior observed when 25 and 55 nm SiO_2_ particles are mixed, as shown in Figure 2f, the concept of effective volume fraction may account for this improved rheology. In a bimodal powder mixture, the smaller particles can occupy the interstitial spaces between larger particles, thereby reducing the void volume and increasing the relative packing density (ϕ_max_) of the system. According to the Furnas binary packing model,^53^ if the large particles first pack to a volume fraction ϕ_*L*_ and the small particles then pack within the remaining voids at ϕ_*S*_, the maximum theoretical packing density can be approximated as

7 $$\phi_{\max}\approx \phi_{L}+\left(1-\phi_{L}\right)\phi_{s}$$

For size ratios similar to those in our 25–55 nm system, the calculated ϕ_max_ versus composition curve reaches a maximum when the volume fraction of the smaller 25 nm particles is close to 50%, as shown in Figure 3a, in agreement with McGeary’s binary sphere packing data.^48^ Here, ϕ_max_ is obtained from DEM-based geometric packing calculations and serves as a structural descriptor independent of fluid-mediated suspension effects. At this ratio, the packing arrangement is most efficient: large particles provide a load-bearing framework while small particles fill interstitial gaps without introducing excessive crowding. A high packing density is beneficial not only because it increases the maximum solid fraction but also because it reduces the void volume in which fluid can be trapped. In systems with excessive porosity, as schematically illustrated in the yellow regions of Figure 3b, a substantial portion of the liquid becomes trapped within these voids, preventing it from contributing to the bulk flow (blue area in Figure 3b) and reducing its capacity to effectively wet particle surfaces.^29^ This entrapment of liquid reduces lubrication between particles, increasing direct particle-to-particle friction, which in turn leads to higher suspension viscosity. In contrast, when porosity is minimized (yellow region in Figure 3c), a larger fraction of the liquid phase remains free to circulate through the particle network, maintaining surface lubrication and facilitating particle rearrangement, thereby reducing viscosity. The Krieger–Dougherty relation^54^

8 $$\eta_{r}={\left(1-\frac{\phi }{\phi_{\max}}\right)}^{-\left[\eta \right]\phi_{\max}}$$

predicts that, for the same solid loading ϕ, a higher ϕ_max_ results in a lower relative viscosity η_*r*_. This directly explains our observation that the mixed suspension shows a nearly constant viscosity across shear rates (Newtonian flow), whereas the monodisperse systems with lower packing densities exhibit strong shear thinning. In the mixed system, the optimized packing suppresses the formation of stress-bearing particle networks even at low shear, while in the monodisperse cases the poorer packing leaves more void space, traps fluid, and promotes stronger particle–particle contacts, increasing viscosity and shear-thinning. This correspondence between packing density theory and our rheological data supports the idea that the geometric arrangement of particles, rather than solely surface chemistry, plays a dominant role in the superior flowability of the mixed-particle suspension.

**Figure 3:**
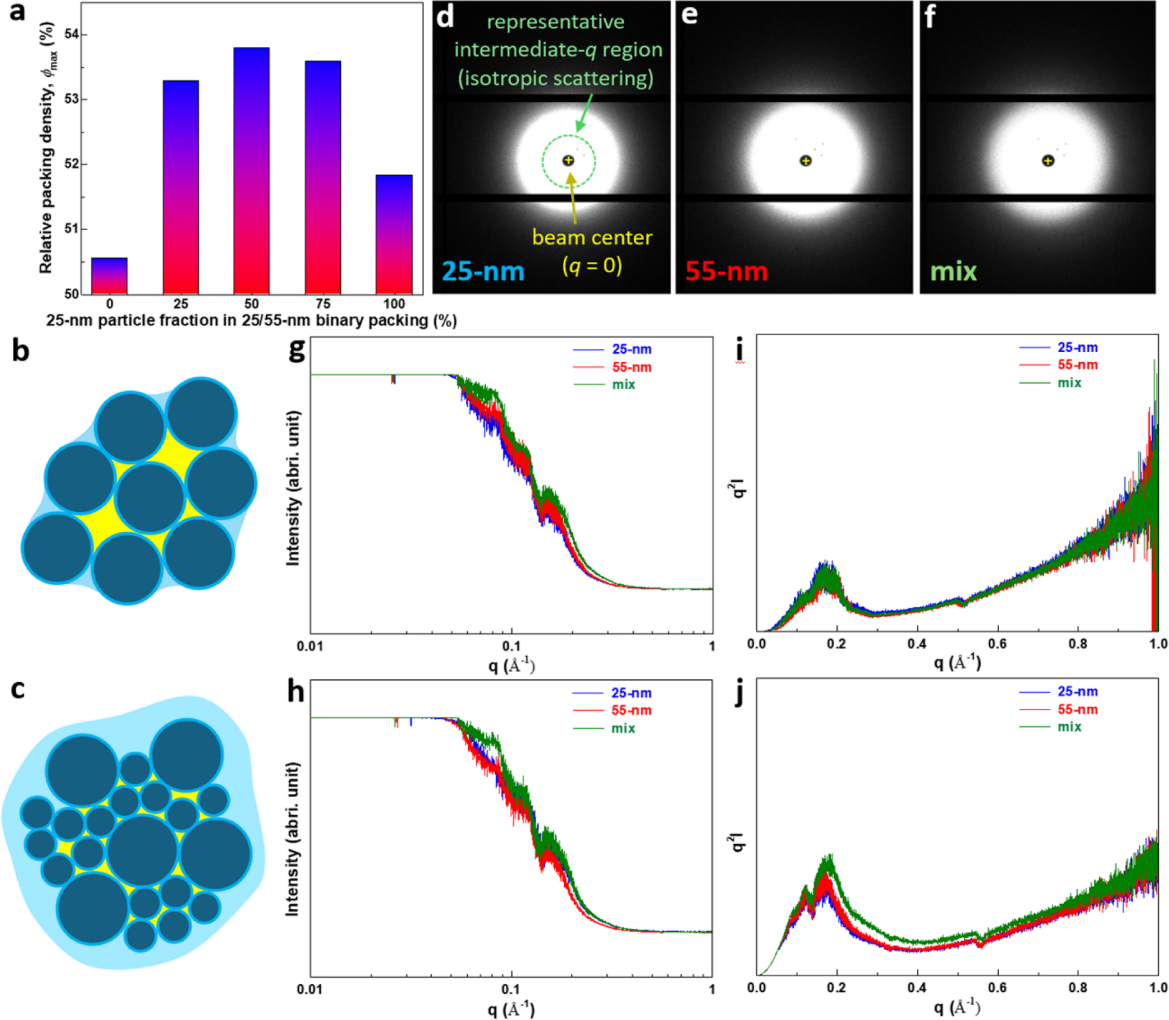
(a) Calculated relative packing densities of mixed SiO_2_ nanopowders at different volume fractions of 25 nm particles, obtained from DEM simulations. Schematics illustrating particle packing configurations with (b) large and (c) small void fractions (yellow regions). Two-dimensional SAXS patterns of aqueous suspensions containing (d) 25 nm, (e) 55 nm, and (f) mixed SiO_2_ particles at a solid loading of 10 wt %, with corresponding one-dimensional SAXS profiles showing scattering intensity as a function of scattering vector (*q*) under (g) static and (i) oscillatory conditions, and their respective Kratky plots in (h) and (j). Note: the dashed circle and annotations shown in panel (d) indicate a representative intermediate-*q* region and isotropic scattering, and are applicable to all three panels (d–f).

Besides the explanation based on the particle packing density, which considers the overall geometric packing efficiency and its influence on suspension viscosity, SAXS was employed to examine the dispersion state and structural organization of SiO_2_ nanoparticles in aqueous suspensions. All SAXS measurements were performed on suspensions at a fixed solid loading of 10 wt % under identical conditions. Such microstructural information provides a complementary perspective on how particle dispersion and interparticle spacing contribute to the observed rheological behavior. Figure 3d–f shows the resulting 2D SAXS patterns for the 25 nm, 55 nm, and mixed particle suspensions. These patterns reveal isotropic and circular scattering, indicating that the particles are randomly oriented and structurally homogeneous in all three systems. Although the 2D patterns appear broadly similar, they are included to confirm isotropy and homogeneity prior to quantitative analysis rather than to highlight contrast. The corresponding 1D SAXS profiles, obtained by azimuthal integration of the 2D patterns, are shown in Figure 3g (static) and 3 h (oscillatory). In SAXS, the scattering vector (*q*) is defined as

9 $$q=\frac{4\pi }{\lambda \sin\left(\frac{\theta }{2}\right)}$$

where λ is the X-ray wavelength and θ is the scattering angle between the incident and scattered beams. This scattering vector is inversely related to a real-space length scale *d* ≈ 2π/*q*. Accordingly, the low-*q* region provides information on large-scale agglomerates or interparticle correlations, whereas the intermediate- and high-*q* regions reflect smaller structural features such as particle size distribution, local ordering, and surface characteristics.

When the suspensions are at rest, the SAXS profiles in Figure 3g show that the low-*q* region remains nearly flat across all three systems, indicating similar large-scale homogeneity. However, in the intermediate-*q* range (≈ 0.05–0.3 Ȧ^–1^), the mixed-particle suspension (green line) exhibits noticeably higher scattering intensity than either monodisperse system, consistent with a broader size distribution arising from its bimodal nature. This higher scattering intensity is a direct consequence of the larger particle size within the mixed system, aligning with principles of particle packing theory. This behavior suggests that the coexistence of small and large SiO_2_ particles produces additional structural correlations and more efficiently fills interstitial voids, reducing local free volume and altering the packing efficiency compared to single-size systems. To further visualize these structural features, the corresponding Kratky plots (Figure 3i, *q* ^2^ *I* vs *q*) are shown. All suspensions display peaks in the range of *q* ≈ 0.05–0.3 Ȧ^–1^, indicating comparable characteristic length scales, while differences in peak shape and intensity reflect variations in distribution breadth rather than fundamental structural length scales.

Upon applying a low-frequency oscillatory perturbation (0.1 Hz, 0.1 Pa), distinct changes appear, as shown in Figure 3h–j. The low-*q* region remains largely unchanged (Figure 3h), confirming that large-scale structural homogeneity is preserved under weak dynamic forcing. In contrast, the intermediate-*q* scattering intensity is significantly enhanced for the mixed suspension, with the intensity consistently following the order: mixed >55 nm > 25 nm. This enhancement is more pronounced than in the static case (Figure 3g), suggesting that oscillatory shear promotes local structural rearrangements and partial disruption of weak agglomerates. The Kratky plots (Figure 3j) magnify this effect, with sharper and more intense peaks observed for the mixed system, indicating enhanced local ordering and interparticle correlations. Overall, these results show that oscillatory forcing accentuates the structural advantages of bimodal packing, providing a microscopic basis for the superior dispersion stability and near-Newtonian rheological behavior observed in the mixed-particle suspension.

### Mixing Strategy vs Dispersant Addition

Previous results demonstrated that mixing larger 55 nm and smaller 25 nm SiO_2_ nanopowders improves their dispersion in aqueous suspension. To evaluate whether this particle-mixing strategy is as effective as or more effective than using a conventional dispersant, we compared it with the dispersion efficiency of PAA-NH_4_. The effect of PAA-NH_4_ adsorption on the surface properties of 25 and 55 nm SiO_2_ was initially examined by measuring their zeta potentials with and without dispersant addition, as shown in Figure 4a (Figure S4, Supporting Information). Upon addition of PAA-NH_4_ (160 mg g^–1^ SiO_2_), the zeta potentials of both powders shifted markedly toward more negative values, and their isoelectric points moved from pH 3–4 to undetectable within the studied pH range, confirming the specific adsorption of PAA-NH_4_ onto the particle surfaces.

**Figure 4:**
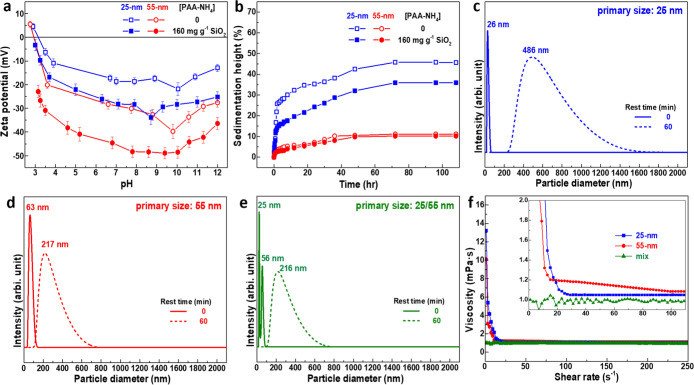
(a) Zeta potentials of 1 wt % aqueous suspensions and (b) sedimentation heights of 10 wt % suspensions for 25 nm, 55 nm, and mixed SiO_2_ particles, with or without PAA-NH_4_ addition (160 mg g^–1^ SiO_2_). Particle size distributions of (c) 25 nm, (d) 55 nm, and (e) mixed SiO_2_ suspensions after ball milling at 180 rpm for 24 h (solid lines) and reagglomeration (dashed lines) following one hour of resting. (f) Viscosity as a function of shear rate for 5 wt % suspensions of 25 nm, 55 nm, and mixed SiO_2_ particles with PAA-NH_4_ addition (160 mg g^–1^ SiO_2_). Note: All aqueous suspensions have an equilibrium pH of 10.

In the sedimentation tests (Figure 4b), adding PAA-NH_4_ causes a reduction in sedimentation height for both suspensions, especially for the one with 25 nm SiO_2_ particles. This suggests that PAA-NH_4_ is particularly effective at reducing the extensive agglomeration that usually occurs in smaller particles, which require stronger electrostatic stabilization to overcome their higher surface-area-driven attractive interactions. Particle size distribution measurements support this finding (Figure 4c): after ball milling, the 25 nm SiO_2_ suspension with PAA-NH_4_ added has a median size of 26 nm, smaller than the 38 nm observed when no dispersant is used (Figure 1c). However, monitoring the suspension over a one-hour resting period (Figure S5, Supporting Information) reveals that reagglomeration still happens, with the median size increasing to 486 nm. Although this is much smaller than the 825 nm measured without PAA-NH_4_ (Figure 1c), it shows that complete prevention of reagglomeration is not achieved, possibly due to an insufficient dispersant dosage, a point that will be addressed in later experiments (Figure 5).

**Figure 5:**
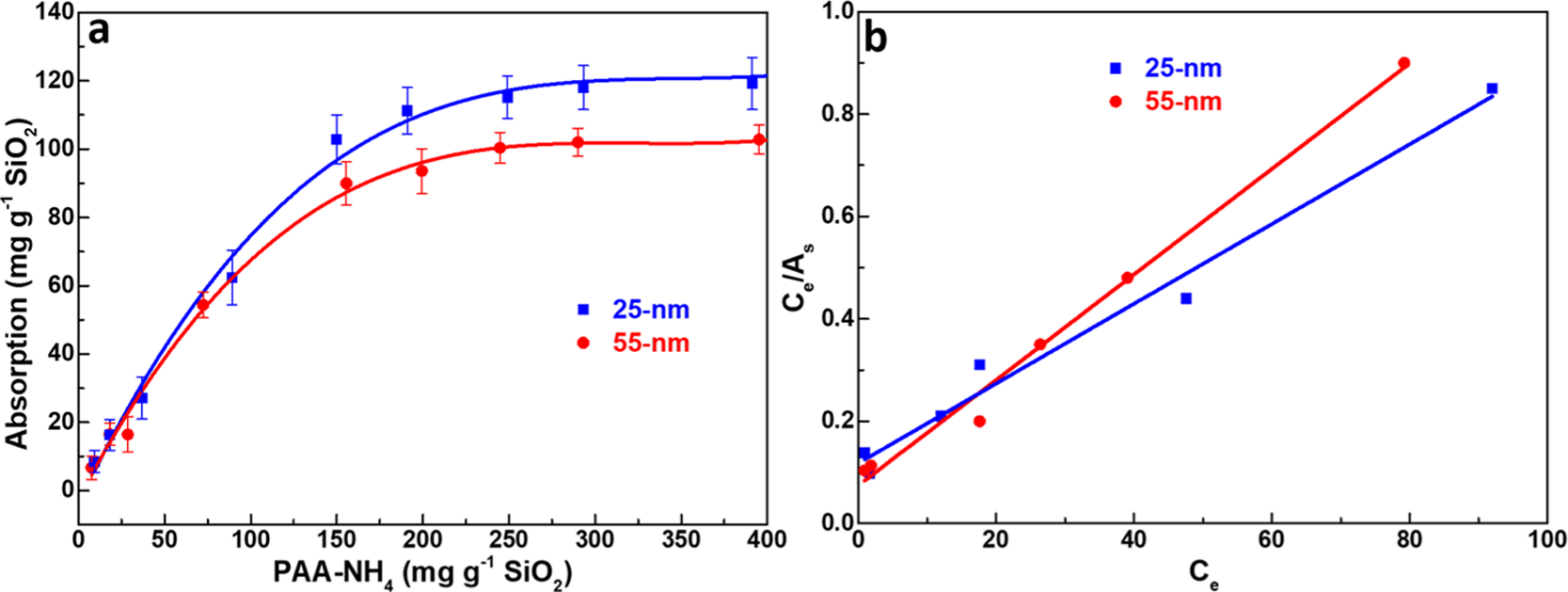
(a) Adsorption amounts of PAA-NH_4_ on 25 and 55 nm SiO_2_ particles as a function of PAA-NH_4_ concentration in suspensions at pH 10. (b) Langmuir plot of (a) using eq 10.

A similar trend is observed for the 55 nm SiO_2_ suspension (Figure 4d). After ball milling, the median particle size is 63 nm, which increases to 217 nm following 1 h of rest. For comparison, the particle size distribution of the mixed-particle system is shown in Figure 4e. After ball milling, a bimodal distribution with median sizes of 25 and 56 nm is observed, which subsequently reagglomerates to a median size of 216 nm. This reagglomerated size is notably smaller than that of the corresponding no-dispersant case (Figure 1e), again highlighting the stabilizing role of dispersant addition. Taken together, these results confirm that PAA-NH_4_ effectively enhances the dispersion of SiO_2_ nanopowders by increasing the magnitude of the zeta potential (Figure 4a), thereby leading to reduced sedimentation and smaller reagglomerated sizes. In the following, the relative efficiency of this chemical dispersant strategy is further compared with that of the particle-mixing approach through rheological studies.

In the rheological analysis, Figure 4f shows the viscosity-shear rate profiles of aqueous suspensions containing 25 nm, 55 nm, and mixed SiO_2_ particles with PAA-NH_4_ (160 mg g^–1^ SiO_2_). Both monodisperse suspensions exhibit pronounced shear-thinning behavior, consistent with the systems without dispersant (Figure 2f), but with lower initial viscosities and lower degrees of agglomeration. Analysis using the Herschel-Bulkley model in eq 6 shows that the *n* values for suspensions with only 25 and 55 nm SiO_2_ particles are 0.78 and 0.82, respectively, indicating improved dispersion compared to those in Figure 2f. In contrast, the mixed-particle suspension exhibits Newtonian flow behavior with an *n* value of 1.01, identical to that observed in the case without dispersant. This comparison demonstrates that particle mixing is inherently more effective than the chemical additive approach in producing a homogeneously dispersed suspension with deagglomerated particles during flow. Such flow stability is particularly advantageous for CMP applications, where residual chemical additives are not only difficult to remove completely but also pose a risk of introducing contamination during the planarization process. While PAA-NH_4_ enhances dispersion to some extent, particle mixing alone delivers superior performance and offers a cleaner, more reliable strategy for CMP slurry design.

### Dispersant Adsorption for Improved Quiescent Dispersion

Although the addition of PAA-NH_4_ improves dispersion to some extent, it does not convert the rheological behavior of either monodisperse suspension from shear-thinning to Newtonian flow, as shown in Figure 4f. To determine whether this limitation arises from an insufficient dispersant dosage, the adsorption isotherms of PAA-NH_4_ on the two SiO_2_ nanopowders were therefore examined (Figure 5). As shown in Figure 5a, the adsorption amount initially increases with added amount and then levels off, consistent with monolayer Langmuir adsorption behavior. Accordingly, the data were analyzed using the Langmuir adsorption model^46^

10 $$\frac{C_{e}}{A_{s}}=\frac{C_{e}}{C_{m}}+\frac{k}{C_{m}}$$

where *C*_e_ is the equilibrium concentration of the free, nonadsorbed PAA-NH_4_ left in the suspension medium, *A*_s_ is the adsorption amount, *C*_m_ is the monolayer adsorption capacity, and *k* is the Langmuir binding constant related to the adsorption energy.^55^ The linear replots of *C*_e_/*A*_s_ versus *C*_e_ (Figure 5b) confirm the Langmuir-type adsorption for both nanopowders. From eq 10, the slopes of the straight lines correspond to 1/*C*_m_, yielding monolayer adsorption capacities of 128 mg g^–1^ and 97 mg g^–1^ for 25 and 55 nm SiO_2_, respectively.

In Figure 5a, the adsorption isotherms indicate that saturation adsorption requires ∼150 mg g^–1^ of PAA-NH_4_ for 55 nm SiO_2_, whereas the 25 nm SiO_2_ demands more than 300 mg g^–1^ due to its larger specific surface area. At an addition level of 160 mg g^–1^, the dosage exceeds the saturation requirement for 55 nm SiO_2_ but falls short of that needed for 25 nm SiO_2_. Notably, the 55 nm suspension maintains shear-thinning behavior even at its saturating PAA-NH_4_ dosage. Based on microstructural considerations, we propose hypotheses for why the shear-thinning behavior persists in the 55 nm SiO_2_ suspension: (i) residual short-range attractions and polymer-mediated interactions (e.g., partial bridging or depletion from free chains) sustain weak, shear-sensitive clusters; (ii) compressible interparticle voids within the packing allow shear-induced rearrangements; and (iii) the adsorbed polymer layers introduce time-dependent interfacial mechanics (such as chain stretching and relaxation). Together, these effects drive a decrease in viscosity with increasing shear rate. For the finer 25 nm SiO_2_ particles, the dispersant dosage is insufficient to fully cover their higher surface area, leaving significant patches unprotected. These exposed surfaces facilitate residual attractive interactions and bridging, further promoting network formation and shear thinning under flow. Consequently, although the addition of PAA-NH_4_ reduces sedimentation and enhances quiescent dispersion stability, it does not sufficiently stabilize the dynamic microstructure of either system to achieve Newtonian behavior. This contrasts with the bimodal particle-mixing strategy, which improves dynamic dispersion primarily through geometric void filling rather than surface adsorption.

### Applications for Planarization

To realize the potential of using the particle-mixing strategy for preparing suspensions for CMP applications, the previously prepared SiO_2_ suspensions were used directly without slurry formulation. In this section, CMP performance is employed as the ultimate benchmark to directly compare the effectiveness of the particle-mixing strategy and the dispersant-based approach under practical polishing conditions. In typical CMP processes, the platen rotation speed is ∼100 rpm; thus, to determine an appropriate polishing duration, the material removal rates (MRR) of different aqueous suspensions were first measured. As shown in Figure 6a, all three suspensions of 25 nm, 55 nm, and mixed SiO_2_ particles reach their highest MRR within the first 15 min of polishing. Therefore, a fixed polishing time of 15 min at 100 rpm was used for subsequent comparisons.

**Figure 6:**
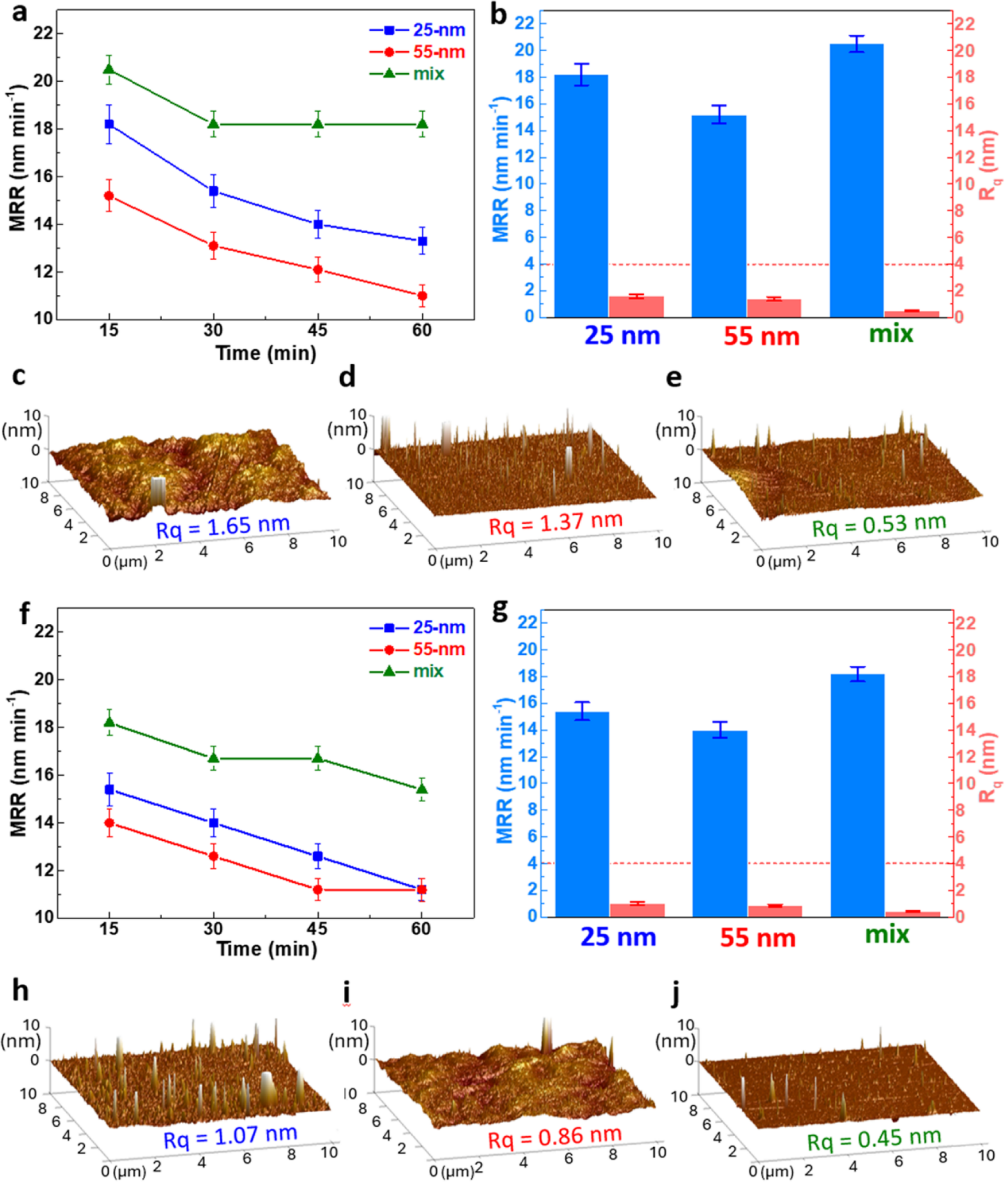
(a,f) Material removal rate (MRR) as a function of polishing time at 100 rpm, and (b,g) surface roughness (Rq) and MRR after polishing at 100 rpm for 15 min using 1 wt % aqueous suspensions (pH = 10) of 25 nm, 55 nm, and mixed SiO_2_ particles. Surface morphologies of Si specimens polished with 1 wt % suspensions of (c,h) 25 nm, (d,i) 55 nm, and (e,j) mixed SiO_2_ particles in the (a–e) absence and (f–j) presence of PAA-NH_4_ (160 mg g^–1^ SiO_2_), at 100 rpm for 15 min.

Under these conditions, Figure 6b summarizes the resulting MRR and surface roughness (Rq) of the polished Si wafers (initial Rq = 4 ± 0.1 nm). Among the suspensions, the mixed-particle system achieves the highest MRR (>20 nm min^–1^), followed by the 25 and 55 nm suspensions. The higher MRR of the 25 nm suspension compared to the 55 nm one can be explained by the formation of larger agglomerates of the 25 nm SiO_2_, which produce greater local stress and thus promote material removal. However, this advantage comes at the expense of surface quality: larger agglomerates also raise the risk of scratches, leading to a rougher surface, as confirmed by AFM images, which show a higher Rq in Figure 6c than in Figure 6d.

In contrast, the mixed-particle suspension simultaneously achieves both the highest MRR (>20 nm min^–1^) and the lowest surface roughness (Rq = 0.53 nm), as shown in Figure 6e. The low Rq value indicates minimal surface scratching, implying that the mechanism behind the enhanced MRR differs from that in the 25 nm suspension. Specifically, the larger 55 nm particles in the mixed system predominantly contribute to effective mechanical removal, while the smaller 25 nm particles occupy interstitial spaces, providing finer-scale abrasion and surface smoothing. This synergistic interplay between coarse and fine particles enables the suspension to deliver high removal efficiency without compromising surface quality. Such dual benefits highlight the unique advantage of particle mixing for CMP applications, it allows high material removal to be achieved through a more homogeneous and stable particle–surface interaction rather than through stress concentration associated with agglomeration. This conclusion will be further supported by the coupled CFD-DEM simulations in Figure 7.

**Figure 7:**
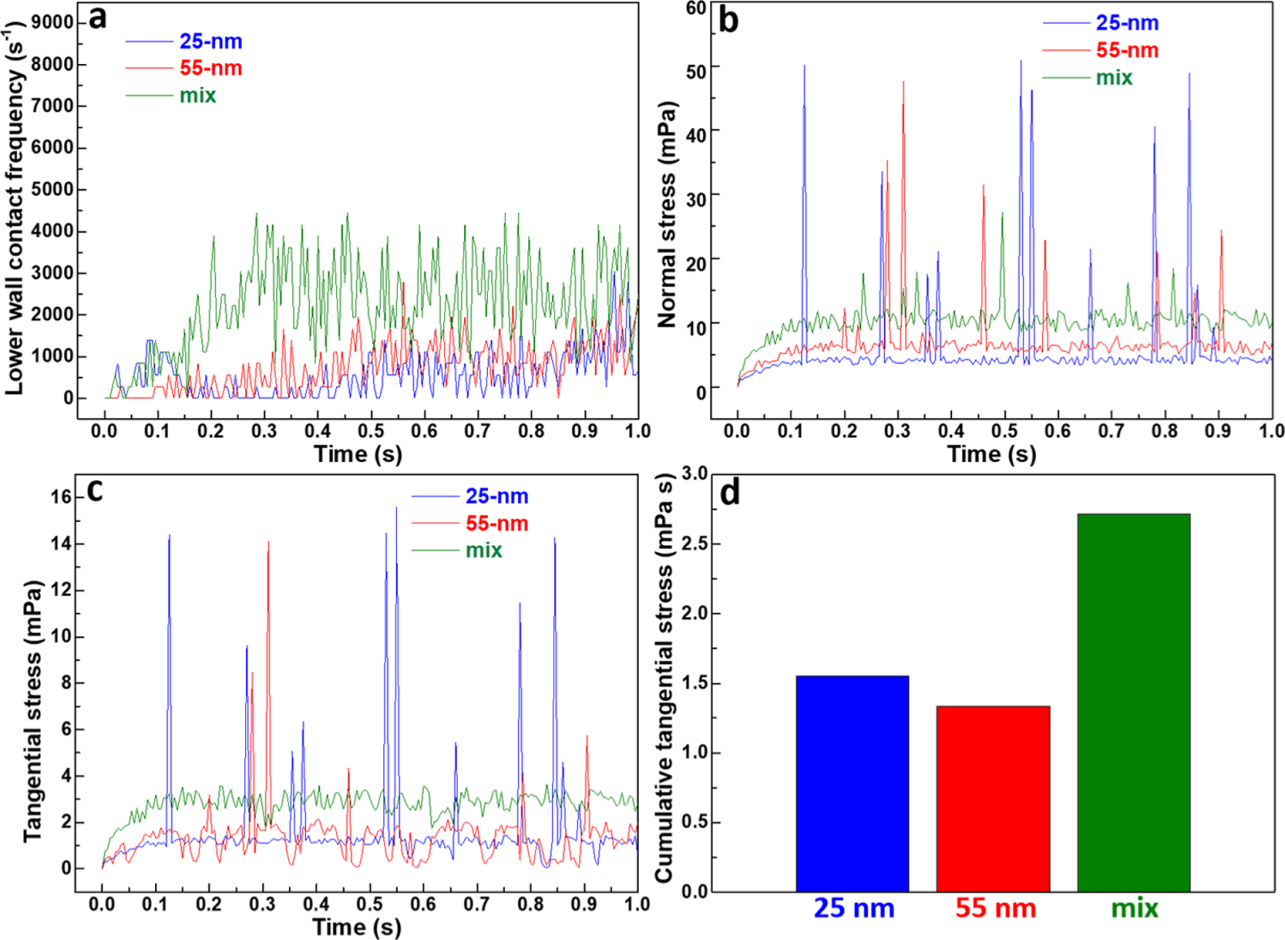
(a) Contact frequency, (b) normal stress, (c) tangential stress, and (d) cumulative tangential stress over a one-second polishing period exerted by aqueous suspensions containing 1 wt % SiO_2_ particles (25 nm, 55 nm, and mixed) at pH 10 under 100 rpm. Note: the simulated duration represents a quasi-steady contact regime used to analyze local particle–surface interaction mechanisms, rather than the full experimental CMP time scale.

To further assess the relative effects of the particle-mixing strategy and the dispersant-adding method on CMP performance, the polishing results of SiO_2_ suspensions containing PAA-NH_4_ (160 mg g-1 SiO_2_) were also evaluated. As shown in Figure 6f, all three suspensions again exhibit their highest MRR within the first 15 min of polishing; thus, the results at 100 rpm for 15 min are compared in Figure 6g–j. For the 25 and 55 nm suspensions, the addition of PAA-NH_4_ leads to smoother surfaces with lower Rq values compared to the corresponding cases without dispersant (Figure 6b). However, this improvement comes at the expense of reduced MRR. The lower MRR is consistent with the improved dispersion and diminished agglomeration of particles caused by PAA-NH_4_, which reduces the localized stress concentrations that otherwise promote faster material removal. For the mixed-particle suspension, the addition of PAA-NH_4_ also results in a decrease in MRR, even though earlier particle size analysis (Figures 1e and 4e) showed no significant reduction in particle size when PAA-NH_4_ is added. This suggests that the decreased MRR is not caused by particle size effects but may instead be linked to other chemical properties of PAA-NH_4_, such as a possible lubrication effect.^56–58^ Meanwhile, the surface quality improves only slightly, with Rq decreasing a little from 0.53 to 0.45 nm (Figure 6j).

Collectively, comparison of Figure 6b–g clearly indicates that, while PAA-NH_4_ modestly enhances dispersion stability, it does not translate into improved polishing efficiency and instead introduces a trade-off between surface quality and removal rate. In contrast, the performance advantage of the particle-mixing strategy, simultaneously achieving higher MRR and lower Rq, is preserved regardless of dispersant addition. These results demonstrate the decisive advantage of particle mixing in CMP slurry design and provide a direct, performance-based validation of the dispersion and rheological analyses discussed in the preceding sections.

### CFD-DEM Simulations for Planarization Mechanisms

It is well understood that effective planarization requires abrasive particles to make sufficient contact with the specimen surface and to apply adequate mechanical stress for material removal. Since these physical and mechanical interactions are difficult to quantify experimentally, we employed a coupled CFD-DEM simulation to investigate the local particle–surface interaction mechanisms during CMP. The objective of this simulation is to capture representative contact statistics and stress characteristics at the slurry–surface interface, rather than to reproduce the full experimental polishing duration. To ensure computational efficiency while retaining the essential physics, a miniaturized and localized model was constructed, consisting of a stationary top plate and a bottom plate rotating at 100 rpm, with 1 wt % SiO_2_ particles suspended in water within a 100 μm gap between the plates. This simplified shear-driven geometry represents a local contact region within the pad–slurry–wafer interface under CMP conditions. Owing to the small characteristic length scale and moderate relative velocities, the flow remains in the laminar regime with a low Reynolds number. Accordingly, the fluid phase was modeled using the physical properties of water, which is appropriate for capturing the local hydrodynamic environment relevant to CMP slurry transport. All simulation parameters and constitutive models employed in the CFD-DEM framework are summarized in Table S1 (Supporting Information).

The resulting simulations for suspensions containing 25 nm, 55 nm, or mixed SiO_2_ particles are summarized in Figure 7. Although the simulated physical time is limited to 1 s, this duration is sufficient to capture statistically meaningful particle-plate contact events and stress distributions. In DEM-based simulations, the extremely small time step required to resolve particle collisions limits the accessible physical time scale; however, the relevant contact statistics rapidly converge to a quasi-steady state. As shown in Figure 7a, all systems reach steady-state contact frequency within 0.2–0.3 s. Notably, the mixed-particle suspension displays a significantly higher frequency of particle-plate contacts compared to either of the monodisperse cases, both of which exhibit similar frequencies. This pronounced enhancement suggests that the mixed system can deliver a denser and more uniform distribution of active abrasives at the surface, thereby increasing the probability of effective surface interactions essential for material removal. These short-time, quasi-steady contact characteristics provide a mechanistic basis for interpreting the experimentally observed trends in material removal rate and surface roughness, rather than serving as a direct time-scale prediction of the full CMP process.

Furthermore, the normal stress generated by abrasive particles (Figure 7b) shows that the mixed-particle suspension exerts the highest stress, followed by the 55 nm SiO_2_ suspension and then the 25 nm system. This hierarchy can be explained by the denser and more effective particle distribution in the mixed suspension, which increases both contact frequency (Figure 7a) and cumulative stress on the plates. Occasional sharp peaks in normal stress are also observed, particularly in the 25 nm suspension, where they are more intense and frequent. These peaks are consistent with transient collision events involving particle clusters or locally dense contact regions interacting with the plates, reflecting the stronger tendency for agglomeration in the finer 25 nm system. In contrast, particle mixing effectively suppresses such agglomeration, leading to smoother stress evolution. The tangential stress, which is directly responsible for material removal, shows a similar trend (Figure 7c). The mixed suspension again delivers the highest tangential stress, consistent with its superior response to normal stress. Interestingly, while the 55 nm suspension generates higher normal stress than the 25 nm system, its tangential stress is nearly indistinguishable from that of the 25 nm suspension. For clearer comparison, the tangential stresses are integrated over the 1 s simulation (Figure 7d). The results demonstrate a pronounced advantage of the mixed system, which produces significantly higher total tangential stress than either of the monodisperse suspensions. The ordering, mixed >25 nm > 55 nm, directly accounts for the experimental MRR trend observed in Figure 6b, linking the enhanced polishing efficiency of the mixed suspension to its superior stress generation and reduced agglomeration.

## Conclusions

This study demonstrates that particle mixing offers a highly effective strategy for enhancing the dynamic dispersion and functional performance of SiO_2_ nanopowders, surpassing the capabilities of traditional chemical dispersants, which are primarily beneficial for quiescent dispersion. Comprehensive analyses combining particle size distribution, TEM, and zeta potential reveal that mixing 25 and 55 nm particles facilitates deagglomeration after ball milling, while mitigating excessive reagglomeration during rest. Although static sedimentation and DLVO analyses do not fully capture the benefits of mixing, rheological measurements clearly establish that the mixed system achieves superior dispersion under flow, transitioning from shear-thinning behavior typical of agglomerated systems to the Newtonian response characteristic of a well-dispersed suspension. SAXS measurements and particle packing density calculations further elucidate the underlying mechanism, showing how the cooperative arrangement of small and large particles promotes enhanced structural organization and stability. The comparison with chemical dispersion via PAA-NH_4_ demonstrates a decisive contrast. While dispersant addition improves particle stability by reducing sedimentation, narrowing particle size distribution, and modifying interparticle forces, it does not achieve the same level of flow uniformity or “dynamic” dispersion efficiency as particle mixing. The practical significance of this advantage is directly reflected in CMP performance. Polishing tests reveal that the powder-mixed suspension simultaneously achieves higher material removal rates and lower surface roughness compared to either monodisperse or dispersant-stabilized suspensions. Coupled CFD-DEM simulations corroborate these results by showing that mixed systems yield denser particle–substrate contact, higher stresses, and more efficient polishing dynamics. These findings establish particle mixing not only as a superior dispersion strategy but also as a scalable, contamination-free route for designing advanced CMP slurries. Beyond CMP, this method provides a versatile framework for enhancing particle dispersions across various fields where chemical additives are problematic, offering a simple yet effective alternative to traditional dispersant methods.

## Supplementary Material





## Notes

Parameters in Table S1 for CFD-DEM simulation; particle size distribution evolution; XPS of 25 and 55 nm SiO_2_; simulated dispersions of SiO_2_ particles at rest and under flow state; Zeta potentials and sedimentation characteristics of SiO_2_ nanopowders (PDF)
CFD-DEM simulation of SiO_2_ particles in an aqueous suspension during flow at 100 rpm (MP4)

## Abbreviations

CMP: chemical mechanical polishing
MRR: material removal rates
SAXS: small-angle X-ray scattering
CFD: computational fluid dynamics
DEM: discrete element method
PAA-NH_4_: ammonium poly­(acrylic acid)
TEM: transmission electron microscope
XPS: X-ray photoelectron spectroscopy
Rq: surface roughness.
