# Supplementary material for: Flow-Induced Dynamic Dispersion in Dispersant-Free Mixed-Oxide Slurry Systems
Source: Langmuir. 2026 Feb 13;42(11):8236–49. doi: 10.1021/acs.langmuir.5c05380 (PMC13019677; doi:10.1021/acs.langmuir.5c05380)
Supplement: Supplementary file 1 [file la5c05380_si_001.pdf]

Supporting Information

Flow-Induced Dynamic Dispersion in Dispersant-Free Mixed-Oxide Slurry Systems

*Yu-An Lin,[1] Feng-Ming Yeh,[1] Bin Hu,[2] Ting-Kai Huang,[2] Hsin-Hsien Lu,[3] Hong Zhong,[2] and Chia-Chen Li[1,]**

[1]*Department of Materials Science and Engineering, National Tsing Hua University, Hsinchu 30013, Taiwan*

[2]*CMP, FUJIFILM Electronic Materials U.S.A., Inc.*

[3]*CMP, FUJIFILM Electronic Materials, Taiwan Co., Ltd*

*Correspondence. E-mail: cc.li@mx.nthu.edu.tw

This Supporting Information includes the following:

Number of pages: 7
Number of figures: 5
Number of tables: 1

Table of Contents:

Table S1. CFD-DEM simulation parameters.

Figure S1. Particle size distributions of monodisperse and mixed SiO_2 aqueous suspensions before and after re-agglomeration.

Figure S2. XPS survey and elemental core-level spectra of 25-nm and 55-nm SiO_2 particles.

Figure S3. Schematic illustration of the dispersion state of SiO_2 particles before and after shear is applied.

Figure S4. Zeta potentials, sedimentation behavior, and visual stability of SiO_2 suspensions with and without PAA-NH_4.

Figure S5. Particle size distributions of monodisperse and mixed SiO_2 suspensions with PAA-NH_4 addition before and after re-agglomeration.

Supplementary Table

Table S1. CFD-DEM simulation parameters.

parameter	units	value or description (55-nm / mix / 25-nm)
Particle density	g cm^{-3}	2.2
Young's modulus	GPa	70
Poisson ratio	—	0.16
Particle number	—	320, 1865, 3410
Surface potential	mV	-39.7, -32.4, -21.8
Restitution coefficient	—	0.9
Static friction coefficient	—	0.3
Dynamic friction coefficient	—	0.3
Liquid model	—	water (Newtonian)
Liquid viscosity	Pa s	0.001
Liquid density	g cm^{-3}	0.998
Normal contact force model	—	Hertzian Spring dashpot
Tangential contact force model	—	Mindlin-Deresiewicz
Drag force model	—	Huilin-Gidaspow
Simulation time	sec	1.0

Supplementary Figures

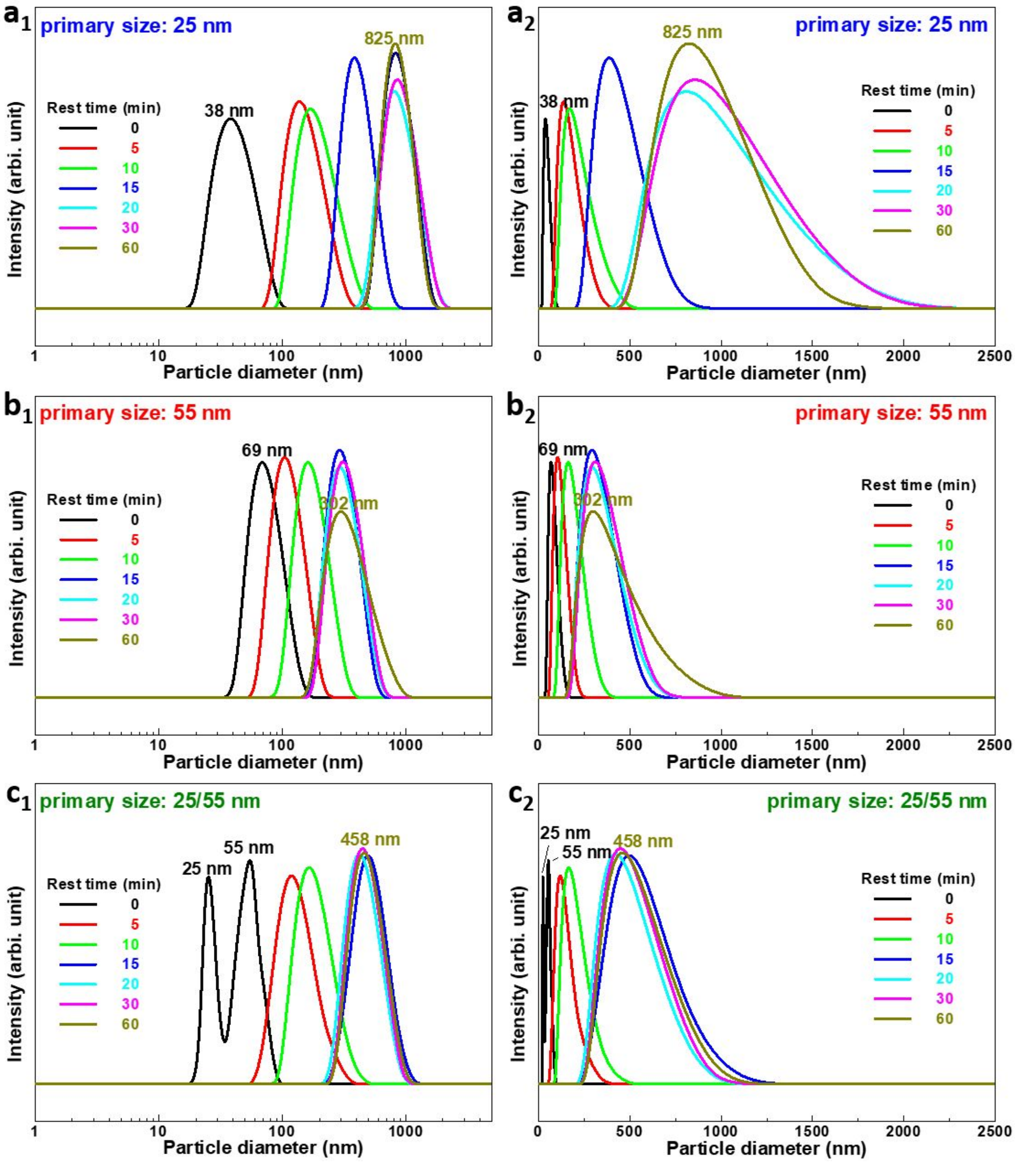

Figure S1. Particle size distributions of 10 wt% aqueous suspensions containing (a_1,a_2) solely 25-nm particles, (b_1,b_2) solely 55-nm particles, and (c_1,c_2) mixed SiO_2 particles, measured after ball milling at 180 rpm for 24 hours (solid lines) and after re-agglomeration upon resting for different periods (dashed lines). All suspensions were maintained at an equilibrium pH of 10.

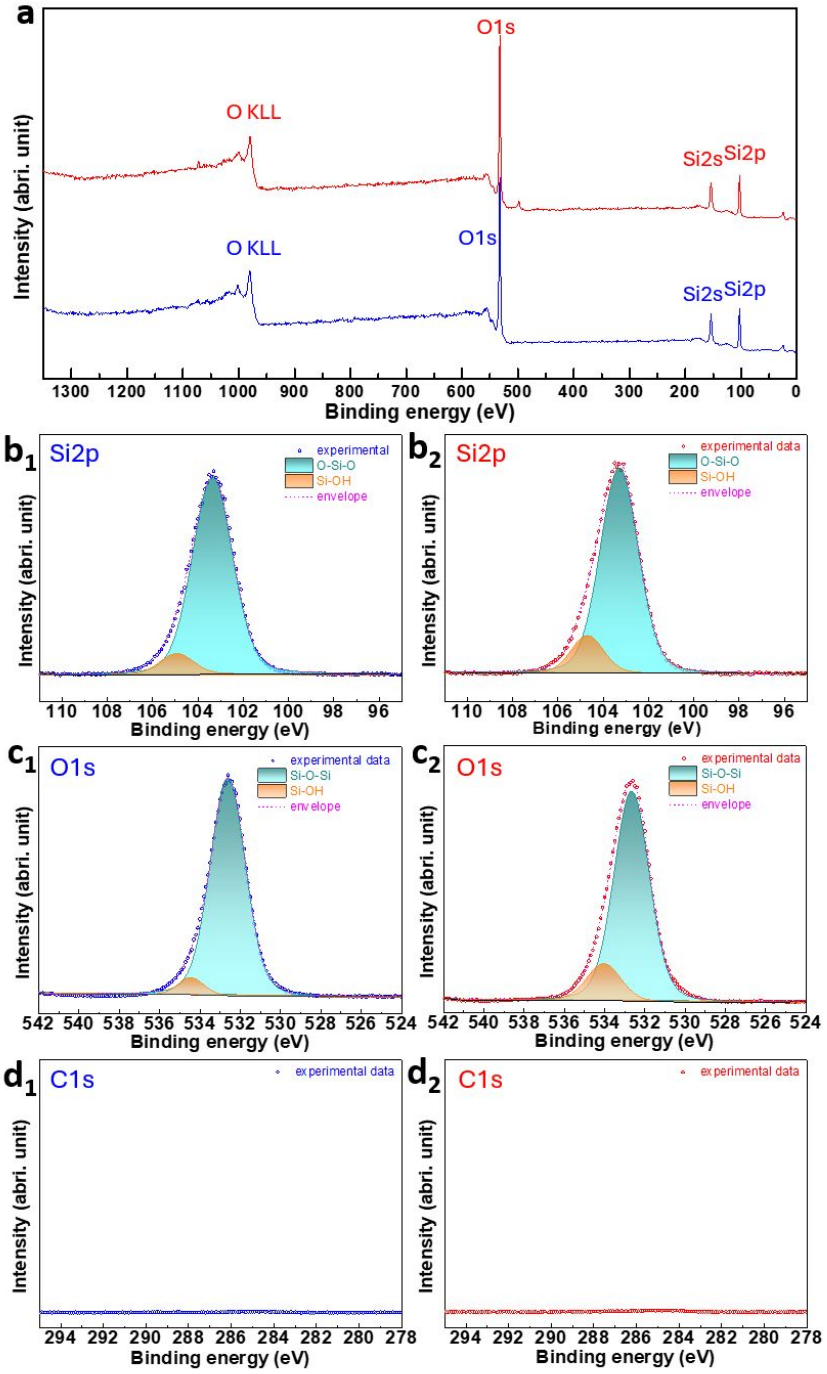

Figure S2. XPS analysis of the 25-nm and 55-nm SiO_2 particles: (a) survey scan; elemental chemical states of (b_1,b_2) Si, (c_1,c_2) O, and (d_1,d_2) C.

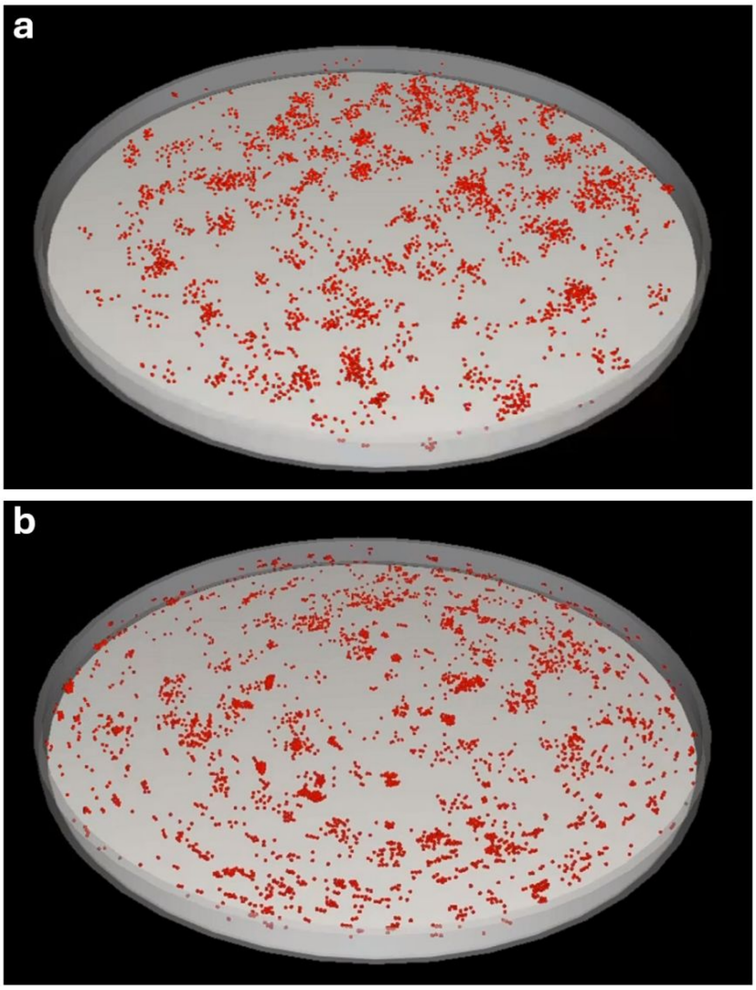

Figure S3. Dispersion state of SiO_2 particles in an aqueous suspension (a) before and (b) after shear stress is applied to flow.

Before shear stress is applied, particles naturally agglomerate in the suspension (Figure S3a). After shearing at 100 rpm (Video S1), the agglomerates are gradually broken apart by shear stress (Figure S3b), which can lead to a reduction in suspension viscosity as shown in Figure 2f in the main article.

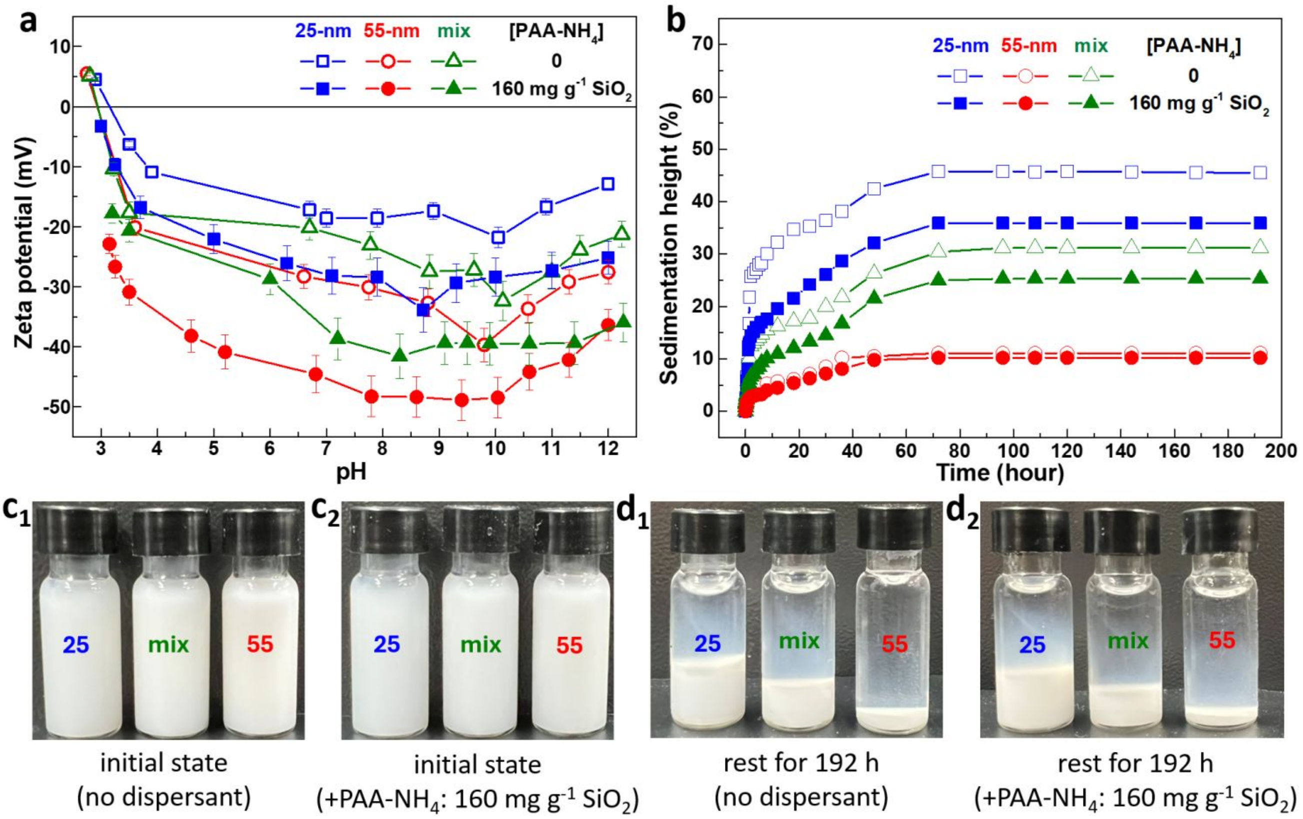

Figure S4. (a) Zeta potentials of 1 wt% aqueous suspensions and (b) sedimentation heights of 10 wt% suspensions containing 25-nm, 55-nm, and mixed SiO_2 particles, with and without $PAA\text{-}NH_4$ addition (160 mg g^{-1} SiO_2). (c_1,c_2) Photographs of suspensions immediately after ball milling and (d_1,d_2) after 192 h of resting, comparing solely 25-nm, solely 55-nm, and mixed SiO_2 suspensions with $PAA\text{-}NH_4$ concentrations of (c_1,d_1) 0 and (c_2,d_2) 160 mg g^{-1} SiO_2. All suspensions exhibit an equilibrium pH of 10.

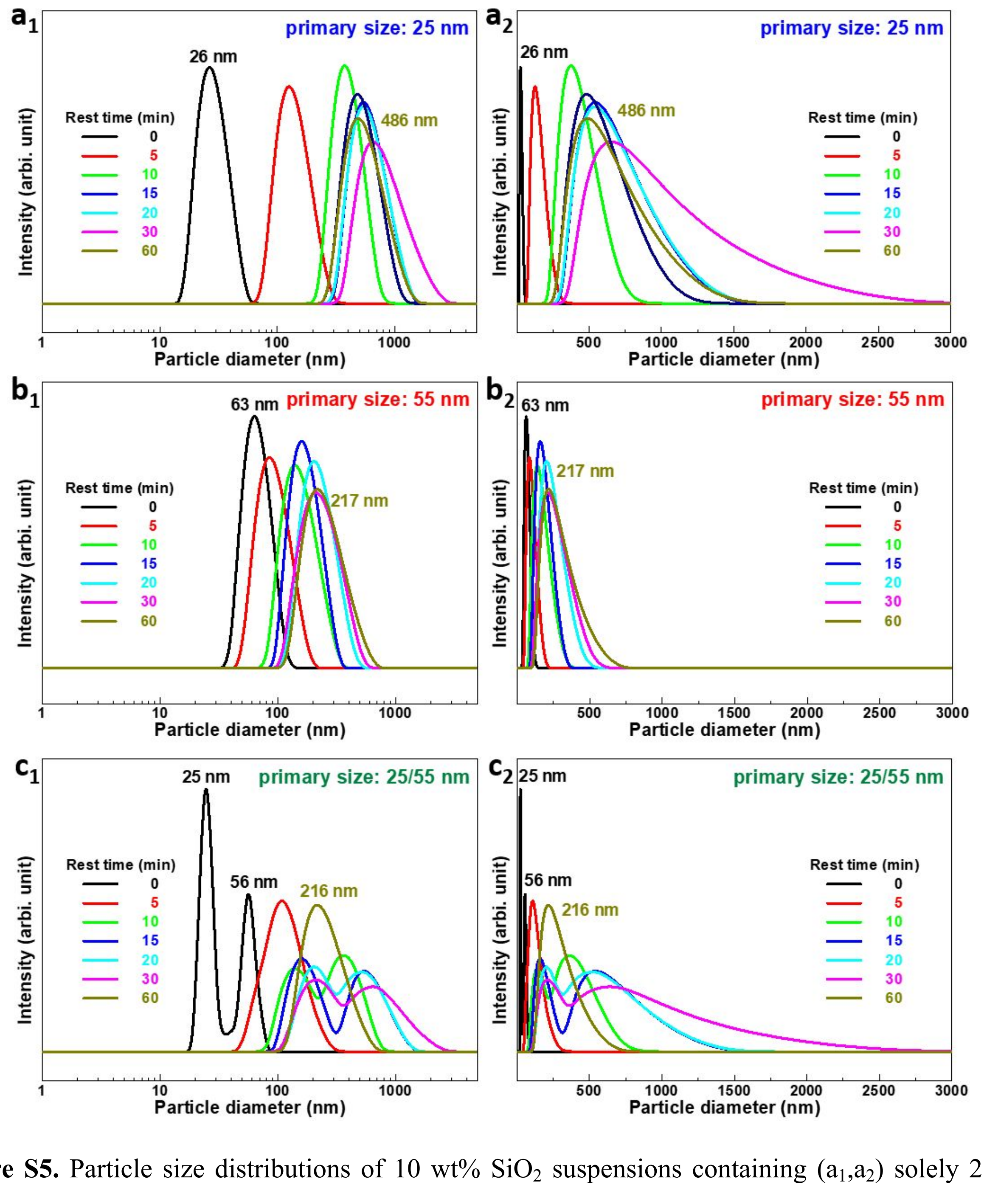

Figure S5. Particle size distributions of 10 wt% SiO_2 suspensions containing (a_1,a_2) solely 25-nm particles, (b_1,b_2) solely 55-nm particles, and (c_1,c_2) mixed particles, with PAA-NH_4 added (160 mg g^{-1} SiO_2). Solid lines represent distributions after ball milling at 180 rpm for 24 hours, while dashed lines represent re-agglomeration after resting for different time periods. All suspensions exhibit an equilibrium pH of 10.
